# Bio-enabled Engineering of Multifunctional “Living” Surfaces

**DOI:** 10.1021/acsnano.3c03138

**Published:** 2023-06-09

**Authors:** Daniel P. Arnold, Sho C. Takatori

**Affiliations:** Department of Chemical Engineering, University of California, Santa Barbara, California 93106, United States

**Keywords:** active matter, bio-enabled engineering, cell membrane, living surfaces

## Abstract

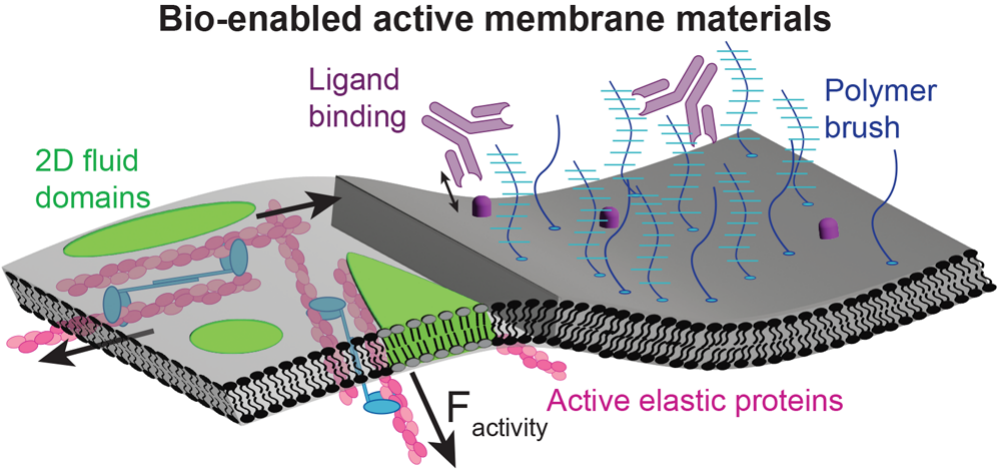

Through the magic of “active matter”—matter that converts chemical energy into mechanical work to drive emergent properties—biology solves a myriad of seemingly enormous physical challenges. Using active matter surfaces, for example, our lungs clear an astronomically large number of particulate contaminants that accompany each of the 10,000 L of air we respire per day, thus ensuring that the lungs’ gas exchange surfaces remain functional. In this Perspective, we describe our efforts to engineer artificial active surfaces that mimic active matter surfaces in biology. Specifically, we seek to assemble the basic active matter components—mechanical motor, driven constituent, and energy source—to design surfaces that support the continuous operation of molecular sensing, recognition, and exchange. The successful realization of this technology would generate multifunctional, “living” surfaces that combine the dynamic programmability of active matter and the molecular specificity of biological surfaces and apply them to applications in biosensors, chemical diagnostics, and other surface transport and catalytic processes. We describe our recent efforts in bio-enabled engineering of living surfaces through the design of molecular probes to understand and integrate native biological membranes into synthetic materials.

Designing complex, multifunctional materials that simultaneously meet several orthogonal design objectives when driven out of equilibrium is a grand challenge for materials scientists and engineers.^1^ Recently, our understanding of nonequilibrium materials has been guided by taking biological inspiration from natural materials in living systems, as part of an emerging field of active matter physics.^2^ Instead of relying upon externally imposed fields and flows, living systems direct collective motion by converting chemical energy into mechanical work at the level of individual constituents. By doing so, they can generate emergent properties that deviate from materials in thermodynamic equilibrium.

In our research, we aim to assemble key active matter ingredients on two-dimensional (2D) surfaces to control nonequilibrium processes and trigger surface activity. Technologies like heterogeneous catalysis, electrochemical reactions, drug delivery, product formulation, and point-of-care diagnostic testing rely on species adsorbing to, being transported across, or reacting at a 2D interface.^3−6^ Engineering soft, multifunctional interfaces with features such as spatially patterned reactivity, self-healing responses to stress, and in-plane species transport may have implications across various disciplines.

Materials scientists often look to biology for inspiration, spurring work to imitate natural material features like structural color, superhydrophobicity, and self-cleaning.^7^ In some cases, it is practical to go a step further and incorporate biomolecules directly into bio-enabled materials,^8^ like enzymes, whose functions are often impossible to reproduce synthetically. In our work, we take the latter approach and integrate purified biomolecular components like lipids, molecular motors, and biopolymers to construct bio-enabled interfacial materials.

Interfacial phenomena are critical to the survival of living systems, and biological cells have developed complex lipid membrane structures to mediate important cellular functions. Conserved across both prokaryota and eukaryota, these soft sheets of amphiphiles self-assemble to form a discrete semipermeable border around the cell.^9^ The structure and organization of cell membranes are complex and heterogeneous, consisting of numerous species of lipids and sterols coupled with transmembrane and lipid-anchored proteins and glycans.^9−11^ The vast assembly of membrane constituents allows cells to generate forces, transport solutes against concentration gradients, and orchestrate site-specific reactions and binding.^9,11,12^ The glycoproteins form a crowded, spatially heterogeneous polymer brush on the extracellular side of the membrane, while the actin and myosin cytoskeleton on the inner side processes adenosine triphosphate (ATP) to produce stresses that reorganize and deform the membrane (Figure 1).^12,13^

**Figure 1:**
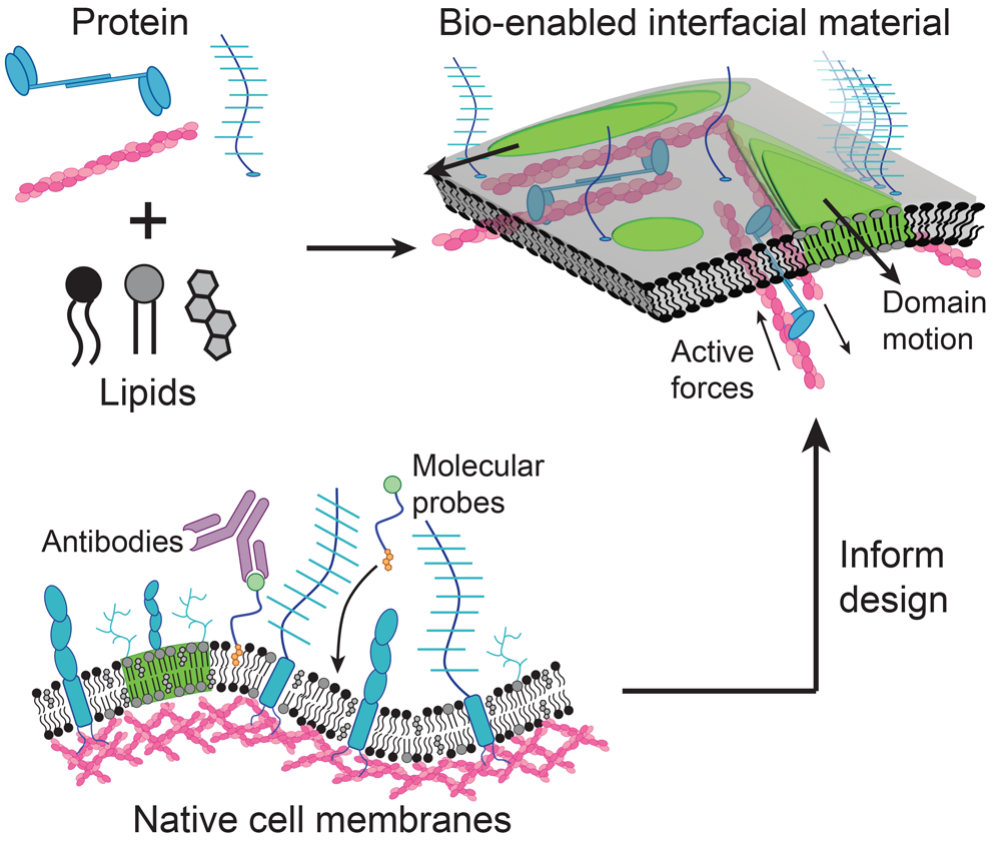
(*Top*) We create bio-enabled interfacial materials by combining lipid membranes with purified proteins like actin and myosin. Force-generating elements such as an actomyosin cortex endow the material with the ability to rearrange lipid and protein constituents using internally driven stresses and flows. (*Bottom*) Our lab has engineered macromolecular sensors to study the biophysical principles that govern the organization of complex membrane materials. Our goal is to apply these principles to design multifunctional “living” surfaces that enable the simultaneous operation of molecular transport, recognition, and catalysis.

Biological membranes are known to achieve exceptional plasticity in response to mechanical forces (∼pN scale) and macromolecular crowding on the cell surface.^14−18^ One key feature that endows plasticity in native cell membranes is the dynamic interplay between the active elasticity of the cellular cytoskeleton and the crowded cell surface. Macromolecular crowding by membrane-bound proteins and sugars on cell surfaces has received considerable attention recently due to its importance in a variety of cellular functions,^17−22^ many of which might be desirable in a synthetic interfacial material. The development of synthetic materials with soft, deformable interfaces like a biological membrane may give rise to programmable surfaces with the ability to remodel and change shape while retaining mechanical integrity.^23^ Yet, important tools to characterize the spatial organization of the cell surface have been missing.^22^ Our lab has recently introduced molecular techniques to study the spatial organization of mammalian cell membranes,^24,25^ which will help us discover ways to incorporate plasticity mechanisms in synthetic active matter surfaces.

We envision the development of multifunctional “living” surfaces that enable the simultaneous operation of molecular transport, recognition, and catalysis. In this Perspective, we discuss our recent work toward understanding and developing these surfaces (Figure 1): (1) present our ongoing work to develop a dynamic 2D composite material of an active elastic network coupled to a viscous fluid membrane and (2) develop techniques to discover the principles of native cell membrane dynamics and organization. We end the Perspective with remaining challenges and frontiers in biomembrane-based 2D complex materials.

## Engineering Bio-enabled “Living” Surfaces

Lipid membranes are capable of large-scale remodeling and shape changes while retaining mechanical integrity.^23^ This plasticity is achieved via the dynamic interplay between active elasticity from the cellular cytoskeleton and the viscous relaxation of the lipid bilayer. Elastic networks formed by biomolecular constituents exhibit characteristics of resilience, toughness, and energy dissipation that are not commonly present in traditional polymer materials that fracture at low strains before dissipation becomes significant.

To these ends, our lab is designing bio-enabled, 2D composite materials that harness biomolecular condensates to alter the overall mechanical behavior of elastic protein networks. In this section, we first discuss prior work on “passive” composite materials before introducing our current work, which incorporates active matter cytoskeletal elements onto bio-enabled surfaces. We then discuss the living nature of these “active” composite materials and the behavior that emerges when mechanical forces are generated internally within the composite systems.

### Design of Viscous–Elastic Composite Materials

In recent years, a substantial body of work has revealed insights into the behavior of 3D viscous–elastic composite materials consisting of liquid–liquid phase-separated droplets embedded within a polymer gel.^26^ In these materials, the liquid inclusions can either stiffen or soften the elastic polymer matrix, depending on droplet size and interfacial tension.^27,28^ Moreover, the matrix can act on the liquid inclusions to suppress and sometimes even reverse coarsening.^29,30^ Composite materials also appear ubiquitously in biology, as the elastic cytoskeleton interacts with both 3D protein and nucleic acid droplets, as well as 2D lipid and protein assemblies on membranes.^31,32^Given that biology has evolved to harness the properties of viscous–elastic composites so effectively, taking a bio-enabled approach may be an effective way to design such materials.

**Figure 2:**
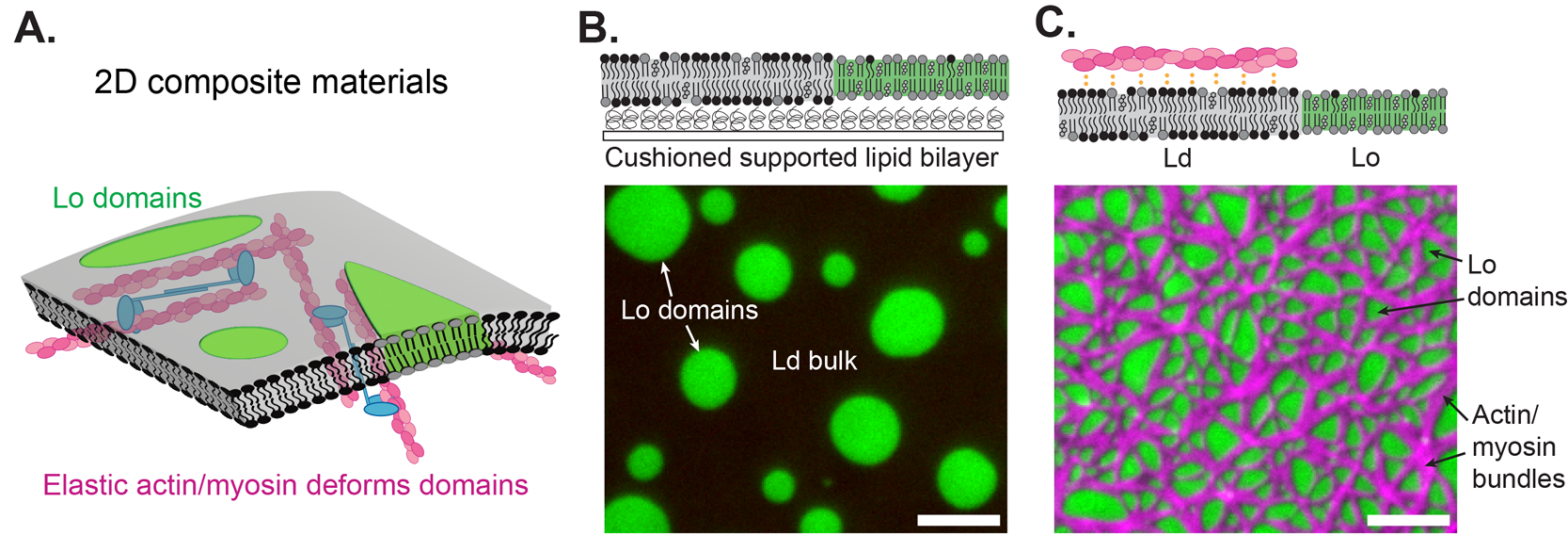
(A) A 2D viscous–elastic composite material is created by coupling molecular motors and filamentous actin (F-actin, magenta) to a phase-separated lipid bilayer. (B) (*Top*) Phase-separated, reconstituted membranes with liquid-disordered (Ld, uncolored) and liquid-ordered (Lo, green) domains. (*Bottom*) Image of Lo domains. (C) (*Top*) A composite viscous–elastic surface decorated with an actin-rich Ld phase and an actin-poor Lo phase. (*Bottom*) Preliminary results of our actin-bound, phase-separated composite membrane. When constrained by F-actin (magenta), Lo domains (green) take on noncircular shapes and do not grow to the same size as unconstrained domains. Scale bars are 5 μm.

In our current work, we study the properties of a 2D composite material composed of molecular motors and filamentous actin (F-actin) coupled to a phase-separated phospholipid bilayer (Figure 2A). We use a cushioned supported lipid bilayer, containing a mixture of saturated lipids, unsaturated lipids, and cholesterol, which phase-separates into a continuous liquid-disordered (Ld) phase and a dispersed liquid-ordered (Lo) phase at room temperature (Figure 2B).^33^ When we adsorb F-actin to the Ld phase of the membrane, we find that the actin acts as a nucleation site for the Ld phase, consistent with prior work on actin-adsorbed reconstituted membranes.^34^ We additionally observe that the F-actin network constrains the Lo domains into noncircular shapes with sharp kinks and corners (Figure 2C).

A comparison between the images in Figure 2B and C qualitatively demonstrates the striking effect of coupling F-actin to the lipid bilayer. We see that the Lo droplets in Figure 2C are unable to grow as large as those in Figure 2B. Moreover, the line tension of 2D phase-separated lipid domains is very low (usually <5 pN) and is dominated by actin elasticity (flexural rigidity 0.2 pN·μm^2^, multiplied by many filaments/bundle), leading to sharp kinks and straight lines in Figure 2C.^12,35^

Our 2D composite material provides a number of advantages when studying interactions between elastic polymers and liquid droplets. The actin bundle mesh size is on the order of microns, approximately the same size as the droplets, rather than on the order of nanometers like in a synthetic, small-molecule polymer gel.^27^ Moreover, the 2D versus 3D dimensionality plays a critical role in governing the overall material properties of the composite. In 3D composites, the liquid droplets have an extra degree of freedom to “escape” out of a local pore created by the polymer mesh. In contrast, our 2D composites lack the out-of-plane degree of freedom, and the liquid domains are physically constrained by the surrounding F-actin mesh. Domain coarsening in our 2D composite is thus dominated by molecular mechanisms such as Ostwald ripening, as collision-based coalescence mechanisms require F-actin relaxation and are kinetically limited in the absence of myosin-driven activity. Because the liquid droplets are incompressible, the embedded droplets will resist mechanical deformation of the entire polymer network more strongly in 2D than in 3D, under external mechanical perturbations like shear deformation. We anticipate that the 2D viscous–elastic composites will inherit a large range of self-strengthening mechanisms. In the future, we will study the mechanical properties of these materials to better understand how embedded liquid membrane domains affect the mechanical properties of the 2D viscous–elastic composite.

### 2D Active Composites and “Living” Surfaces

The bio-enabled nature of our 2D composite materials described in Figure 2 motivates the use of molecular motors to create active or “living” materials. We use the term “active” to refer to a molecular system that converts chemical energy, in the form of ATP, into mechanical motion. The cellular cytoskeleton, where motors like myosin and kinesin generate mechanical forces against microtubules and actin,^12^ is an active network that allows living organisms to operate out of equilibrium. Myosin motors enable F-actin to act as a dynamic network of filaments that can reorganize when perturbed by external or internal stimuli.^36,37^ Many studies have used purified cytoskeletal components to internally generate stresses in active materials systems.^2^

Molecular motors and actin filaments inside living cells can exert forces on the cell membrane, allowing cells to bend, deform, and stretch their surfaces.^38^ Recent experiments have begun to address this important coupling between the active forces and membrane dynamics in reconstituted systems. Kinesin-driven microtubules can generate protrusions in reconstituted lipid vesicles and drive both fission and fusion of liquid–liquid phase-separated droplets.^39,40^ Moreover, Vogel et al. showed that actin and myosin adsorbed to a lipid bilayer can drive short-lived shape changes in lipid membrane domains and can trigger sporadic domain fusion and fission events.^41^ However, despite these advances, the mechanisms by which nonequilibrium forces modulate the dynamics of fluid membrane surfaces remain largely unknown.

**Figure 3:**
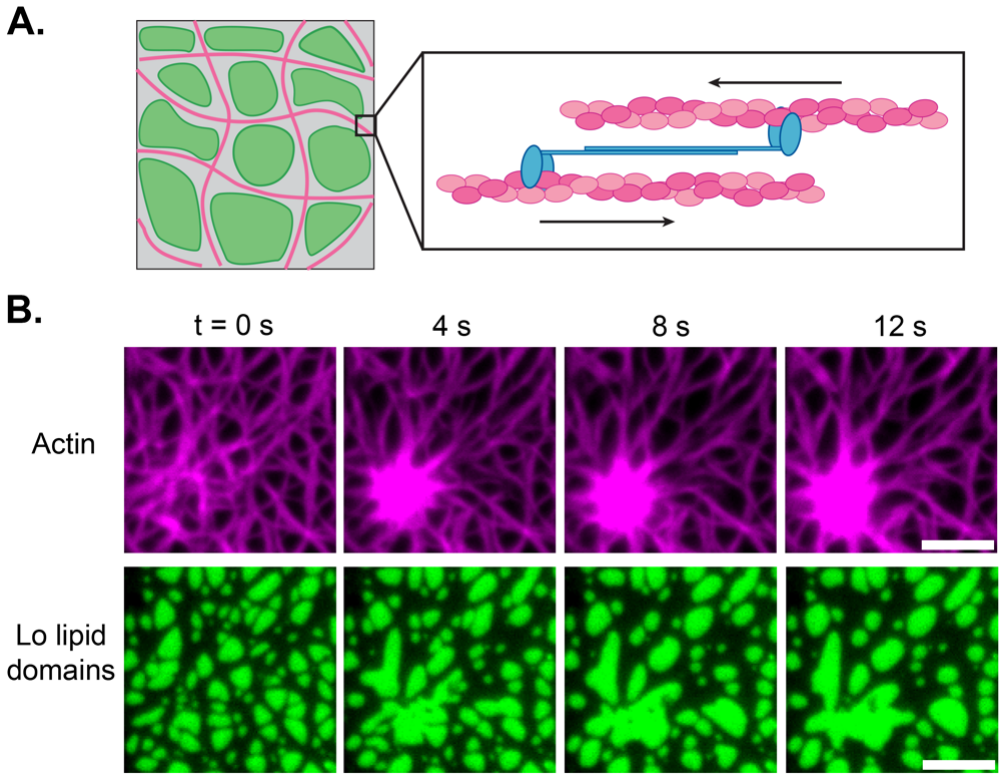
Actin–myosin activity remodels membrane domains and facilitates coalescence. (A) (*Left*) Schematic of F-actin (magenta) adsorbed to a phase-separated membrane, as described in Figure 2B. We localized the F-actin to the Ld (gray) phase. (*Right*) When ATP is added, myosin II (blue) contracts and generates forces on F-actin. (B) ATP is introduced at *t* = 0, causing the actomyosin network to contract. As the actin network contracts, active flows generated along the membrane cause the Lo domains to rapidly grow and change shape. Scale bars are 5 μm.

Inspired by these previous examples of active networks coupled with embedded condensates, we used ATP to cause the actomyosin gel bound to our composite membrane to contract (Figure 3A). As the myosin contracts, F-actin is pulled into asterlike points, and the liquid-ordered domains rapidly mix and fuse (Figure 3B). Unlike the sporadic fission and coalescence reported by Vogel et al. and Adkins et al.,^40,41^ we observe rapid coalescence directed by an apparent radial flow of actin drawing the domains inward. The speed and deterministic nature of the domain coalescence are interesting when compared to existing literature describing lipid domain coarsening in passive systems, in which domain size *a* grows in time as *a* ∼ *t*^1/3^, consistent with standard models for both Ostwald ripening and coalescence.^42,43^ In future work, we will study the activity-driven acceleration of domain coarsening beyond the kinetics observed in passive systems. This future work may inform the design of 2D multifunctional materials with internally driven spatial patterning.

In summary, by integrating active matter constituents into multicomponent fluid membranes, we achieve control over the phase separation and material properties of the membrane surface. Dynamic softening and rearrangement of surface species make our actin and lipid membrane composite material an ideal system for the future study of multifunctional interfacial materials.

## Developing Tools to Characterize Cell Membranes and Design Multifunctional “Living” Surfaces

The plasma membrane of living cells mediates important chemical and physical processes, such as the selective transport of ionic species, catalysis and recognition of biomolecules, and sensing of specific chemical species in solution. Many of these properties are highly desirable in engineered materials, and the integration of key components of the cell membrane into synthetic material surfaces may enable applications in biosensors, chemical diagnostics, and other surface transport and catalytic processes. The successful realization of this technology would first benefit from an improved understanding of the spatial organization of the native cell membrane, including the glycocalyx, a dense coating of membrane proteins and glycans covering the cell surface.^9^ However, we currently lack quantitative methods to obtain a detailed, mechanistic understanding of plasma membrane organization and the biophysical interactions that govern macromolecular binding on crowded cell surfaces. To address this gap, we engineered macromolecular sensors to study the molecular-to-mesoscale interactions that regulate plasma membrane organization.

### Engineered Macromolecular Sensors Provide Insight into Live Cell Membrane Organization and Heterogeneity

Depending on the application, ligand binding on synthetic surfaces may be desirable (as in the case of chemical catalysis) or harmful (as in the case of fouling). Polymer brushes adsorbed to surfaces sterically repel adsorbing ligands in a height-dependent fashion,^44^ which may provide a useful “filter” for bio-enabled interfaces. The glycocalyx behaves like a polymer brush, which swells away from the membrane due to steric and other repulsive interactions between neighboring proteins.^25,45,46^ Thus, engineering protein or polymer brush architectures onto a bio-enabled surface requires a deep understanding of the complex role of macromolecular crowding at biological interfaces. To these ends, we have designed tools to study the organization of surface proteins and sugars on live cells, with the ultimate goal of designing engineered materials with optimized membrane morphology and ligand binding.

Macromolecular crowding on the cell surface can be quite significant and is capable of deforming both reconstituted and native mammalian cell membranes into a tubular morphology, ultimately leading to membrane fission in extreme cases.^17,18,47^ Studies on reconstituted membranes have also found that crowding can prevent soluble ligands like antibodies from binding to the surface.^25,48,49^ The reduction in antibody binding due to membrane crowding near the surface may be problematic in drug delivery applications, since large therapeutic drugs like antibodies experience an energy penalty to bind to target antigen receptors buried within the dense cell surface brush.^25,46,50^ We recently capitalized upon this brush-mediated antibody repulsion and developed strategies to characterize spatial heterogeneities in crowding on both reconstituted and native plasma membranes using antibody binding.^24^

**Figure 4:**
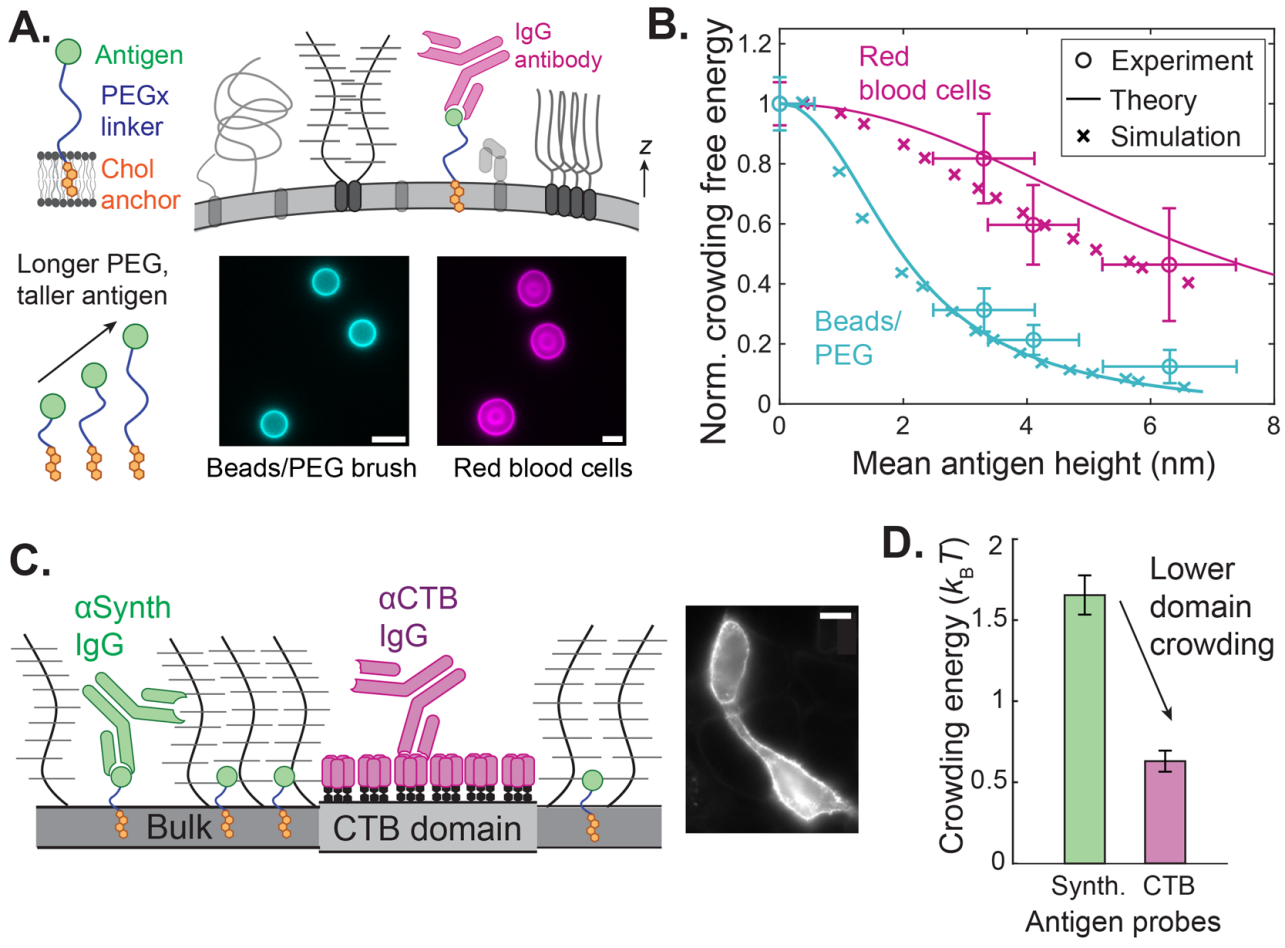
Molecular probes characterize the spatial organization of reconstituted and native plasma membranes. (A) Synthetic antigen sensors composed of a cholesterol anchor, variable poly(ethylene glycol) (PEG) linker, and small antigen are introduced to crowded membrane surfaces. Immunoglobulin G (IgG) antibody binding is measured at varying heights above the surface, with the mean height being controlled by the length of the PEG linker. Fluorescence images of (*left*) supported lipid bilayers on silica beads and (*right*) human red blood cells (RBCs). Scale bars are 5 μm. (B) Normalized surface free energy due to crowding is plotted as a function of the mean height of each sensor on both reconstituted membranes and human RBCs. Molecular dynamics (MD) simulations and polymer brush theory agree with the experiments. (C) IgG antibodies report lateral heterogeneities in crowding on native HeLa cell membranes containing synthetic antigen (green) or cholera toxin B (CTB, magenta) probes. CTB forms distinct surface domains from the bulk membrane, where synthetic sensors predominate. A representative micrograph of CTB bound to a HeLa cell is displayed on the right. Scale bar is 10 μm. (D) Free energy due to crowding (Δ*U*), as reported by the synthetic and CTB antigens for HeLa cells. The CTB domains on the cell membrane experience stronger relative antibody binding and are thus less crowded than those on the bulk membrane. Adapted with permission from ref (24). Copyright 2023, The Author(s), Published by Springer Nature under the terms of the Creative Commons CC BY license.

Due to the strong gradients in membrane-proximal glycocalyx density on certain species of cells (∼10 nm on human red blood cells (RBCs), for example), it was previously very difficult to accurately measure crowding heterogeneites on such short length scales without resorting to destructive techniques like cryo-electron microscopy^51^ and detergent-resistant membranes.^52^ Atomic force microscopy (AFM) has been effectively used to characterize endothelial glycocalyx stiffness and spatial topography, but it primarily captures glycocalyx behavior on length scales of ∼100 nm, without nanometer-scale resolution near the surface.^53^ The surface forces apparatus (SFA) is useful for measuring forces between brush-grafted reconstituted lipid bilayers with nanometer precision,^54^ but it is unable to make measurements on native cell membranes, as they cannot easily be spread on its mica surface. Existing molecular techniques have lacked significant spatial resolution and were unable to measure cell membrane crowding and organization at 5–20 nm distances away from the membrane surface, where many of the important ligand–receptor interactions occur.^20−22,25^ Therefore, we adapted the methods used by Takatori and Son et al.^25^ to engineer antigen probes that insert into the lipid membrane with adjustable antigen heights. These probes consist of a cholesterol anchor, which inserts into a native lipid membrane, connected to a small antigen by a variable poly(ethylene glycol) (PEG) linker (Figure 4A). Thus, we have created a synthetic “receptor” with, due to the variable PEG molecular weight, tunable height at which antibody binding occurs.

Using experiments with our molecular probes, in conjunction with molecular dynamics (MD) simulations and analytical theory, we showed that antibody binding decreased as a function of distance from both reconstituted membranes with a repulsive PEG brush and native RBC membranes (Figure 4B). We characterized this reduction in binding as a free energy barrier to binding, due to brush crowding, proportional to the osmotic pressure experienced by a colloid adsorbing to a brush.^24,44^ Thus, we find that antigen location strongly influences antibody binding on crowded cell membranes, with the strongest gradients occurring within a few nanometers of the membrane. The results in Figure 4B highlight the high degree of sensitivity offered by molecular probe-based techniques for measuring crowding, with the ability to make precise measurements of energy penalties and osmotic pressures over a 10 nm distance without fixing, freezing, or destroying the cell. These techniques may be used to control the material properties and spatial organization of engineered membranes decorated with a biopolymer brush of receptors, enzymes, and protein pumps.

Our molecular probes may be used to measure membrane-orthogonal crowding heterogeneities on both live biological membranes as well as synthetic membrane materials and devices. Given the existence of membrane-proximal antibody drug targets,^46^ we expect our crowding measurements to provide another tool for screening and optimizing targets for antibodies and other biologic drugs. In addition to drug delivery, we envision using crowding on complex interfaces as a tool to actuate molecular sensing and recognition. One can be functionalized with large mucin glycoproteins to build a biomolecular polymer brush with a prescribed mesh size and electrostatic charges. The brush will thus trap species with specific sizes and chemistries such as foreign contaminants or analytes of interest. In this way, the biomolecular polymer brush acts as a semipermeable filter that traps certain species while allowing other molecules that do not interact with the mucin to penetrate freely. Incorporating membrane-orthogonal brush heterogeneity into surface-reactive interfacial materials may also provide an opportunity to colocalize multiple reactive or catalytic species on the surface, while varying their relative ligand affinities by tuning only their height.

### Molecular Probes Provide Insight into Designing Phase-Separated “Living” Surfaces

In addition to exhibiting complex membrane-orthogonal glycocalyx organization, cell membranes are composed of many different lipid and protein components, which can self-assemble into complex structures to perform higher-order functions.^11,55^ Yuan et al. demonstrated that membrane-bound FUS LC, an intrinsically disordered protein known to form phase-separated liquid droplets in 3D suspension, can also phase-separate into 2D domains on giant unilamellar vesicles (GUVs).^56^ Along these protein-rich domains, the attractive interprotein interactions can bend the membrane into concave tubule structures, which adopt a pearled morphology in response to increased salt concentration. As protein clustering can give rise to rich and complex physical behavior, it is important to understand the spatial distribution of surface species on native cells, so that we may better harness these features when designing bio-enabled “living” surfaces.

Giant plasma membrane vesicles (GPMVs) isolated from native membranes are often used as a model system to study the heterogeneous distribution of cell membrane components.^57^ Studies on GPMVs have shown that ordered lipid domains tend to exclude transmembrane proteins, particularly those with bulky extracellular domains, leaving only glycosylphosphatidylinositol (GPI)-anchored proteins behind.^58,59^ However, like reconstituted GUVs, GPMVs when quenched, typically phase-separate into macroscopic domains, which are not representative of the smaller and more heterogeneous lipid and protein clusters present on live cells. A deeper understanding of the active mechanisms by which cells maintain these small, nonequilibrium domains via the cytoskeleton may better inform the design of bio-enabled living materials like those we developed in the previous section.

We recently addressed the gap between the studies on GPMVs and the nonequilibrium effects of transient and small protein clusters on the crowding landscape of native mammalian plasma membranes. We applied spatially selective antigens to the surface of human cervical cancer HeLa cells and measured the crowding penalty for each species (Figure 4C).^24^ We found a ∼65% reduction in crowding energy on cholera toxin B (CTB) antigens compared to our synthetic cholesterol-based antigens (Figure 4D). CTB binds to the ganglioside lipid GM1 and is known to oligomerize on the membrane, recruiting other ordered lipids and sometimes precipitating lipid phase separation.^60^ However, these aggregates tend to form on the nanometer scale and cannot be distinguished from the bulk via microscopy, requiring the use of spatially targeted antigens for effective measurement.

The reduced crowding we observe near CTB further supports the hypothesis that bulky proteins are excluded from ordered domains on native cell membranes as well as GPMVs. Our results further suggest the importance of nonequilibrium effects, like mechanical activity from the cytoskeleton, in maintaining a complex spatial distribution of surface species on the plasma membrane. Endowing bio-enabled materials with similar internally driven mechanisms to maintain and dynamically alter composition heterogeneity may give rise to artificial surfaces that pattern or internally mix surface species, just as cells do.

Taken together, these results suggest that the biomolecular makeup of proteins and lipids on synthetic membrane materials can be used to tune both the magnitude of crowding and the spatial organization of surface-bound enzymes, receptors, and pumps. Spatial-patterning of crowding also offers the possibility of protecting certain surface species from ligand binding while leaving clear access to other soluble species. In this way, the protein and sugar brush decorating the surface may be engineered to allow specific macromolecules to pass through while keeping others out, analogous to the semipermeable barrier performance of the lipid bilayer against ionic species and other small molecules. Furthermore, the coupling of membrane surfaces with an underlying actin cytoskeleton may facilitate a nonequilibrium distribution of surface species on native membranes, and this principle could be used to control engineered surfaces driven away from thermodynamic equilibrium.

## Challenges and Outlook

We believe that the framework of designing tools to study native biological membranes, and then translating these principles into bio-enabled surfaces, will advance the development of multifunctional interfacial materials with complex functionalities. In this section, we briefly discuss outstanding challenges in each approach and then conclude by discussing potential applications of bio-enabled active matter surfaces.

A 2D actomyosin network adsorbed to a phase-separated lipid bilayer provides a rich model system for engineered surfaces owing to the physics conferred by its two-dimensionality and the activity-driven structural modification. Quantifying the mechanical properties will be critical to understanding the strengths and limitations of composite materials constructed in this way. One challenge is the difficulty of conducting interfacial measurements such as rheology on complex 2D interfaces, which is a challenge that exists in general for all materials with interfaces. In particular, it will be important to understand the impact of the phase-separated 2D lipid domains on the overall composite stiffness and whether the depth to which the bilayer is quenched below the miscibility line plays a role in determining the material properties. Further measurement of composite network stiffness before and after adding activity will also provide insight into the effectiveness of activity in softening a composite material. Measuring spontaneous actin fluctuations both before and after triggering activity may provide clues regarding the impact of local stiffness on actin behavior. The ability to internally and dynamically control interfacial stiffness through activity may prove valuable for designing lipid nanoparticles and other liposome-based technologies, as it provides a means of toggling suspension rheological properties and particle binding affinity.

Utilizing molecular probes to characterize native membrane properties, and translating those properties into useful materials, also still faces a number of outstanding challenges. While the size of synthetic probes such as cholesterol-PEG-FITC is relatively easy to tune, it is a greater challenge to identify suitable laterally heterogeneous membrane probes, such as the cluster-forming CTB, to provide useful information about cell-surface heterogeneities. Measuring antibody binding to GPI-anchored proteins might provide a superior understanding of crowding in ordered membrane domains, as these proteins are strongly associated with raft-like lipid domains.^58,59^ Other molecular probes, such as the sensors described by Hsu et al., which measure membrane tension on live cells,^61^ promise exciting experiments studying the material properties of live cell membranes. Dynamically measuring the distribution of stresses in the plasma membrane during mechanically perturbative processes like cell adhesion and movement and correlating these stresses with surface protein distributions may offer further insight into how membrane material components can be dynamically redistributed using flow.

Our aim is to confer upon synthetic membrane surfaces dynamic properties that mimic those of native cell membranes, such as selective molecular exchange, biomolecular recognition, and enzymatic activity. The combined use of theory, simulation, and experiments is needed to engineer multifunctional “living” surfaces that integrate the dynamic programmability of active matter with the molecular specificity of biological membranes. An ability to recognize and transport specific molecular species based on biophysical surface modulation may enable the continuous operation of biosensors, chemical diagnostics, gas exchangers, and other surface transport and catalytic processes.

Surface diffusion of adsorbed reactants or intermediates is often a rate-limiting step in industrial heterogeneous catalysis,^3^ and this motivates the development of internally mixed catalytic surfaces. Several authors have addressed this problem using co-adsorbed species like phosphonic acid or nitric oxide to reduce the translational entropy of surface-adsorbed intermediates and thus accelerate reactions like carbon monoxide (CO) oxidation over a rhodium catalyst, or acetylene conversion to benzene over a palladium catalyst, respectively.^62−64^ In addition, seminal work by Gerhard Ertl, who won the Nobel Prize in Chemistry in 2007, showed that, during CO oxidation on a platinum catalyst, CO and oxygen enrich in distinct, spiral-shaped domains, with reactions occurring at domain interfaces.^65,66^ Thus, internally-driven mixing of reactive surface species may be highly desirable in certain surface-catalyzed industrial reactions, with the soft phase-separating system we present here providing an exciting opportunity to address this gap.

While a soft, bio-enabled interface is chemically very different from a metal catalyst, it may serve as a dynamic, “living” platform for reactions catalyzed via membrane-adsorbed enzymes. Self-driven biopolymers such as actin and microtubules can generate turbulent-like surface flows that mix the reaction surface vigorously, accelerating the chemical reaction to proceed at conditions corresponding to effective temperatures tens or hundreds of times larger than ambient conditions, thus overcoming diffusion limitations and maximizing the overall reaction rate. In biology, ensembles of enzymes, such as horseradish peroxidase and glucose oxidase, associate to form multienzyme complexes (MECs) that can quickly catalyze multistep reactions due to their proximity.^67^ On membranes, it has been shown that the relative ratios of membrane-bound kinases and phosphatases located in close proximity can either activate or deactivate downstream biochemical reactions and signaling pathways that trigger macrophage phagocytosis.^68^ Furthermore, MECs have been reconstituted on nanoparticles and other surfaces in vitro to create synethic biocatalytic devices.^67^ Using internally mixed, “living” fluid interfaces to mix surface-adsorbed enzymes so that they can more quickly assemble into reactive complexes may significantly enhance reaction rates on reconstituted catalytic surfaces.

**Figure 5:**
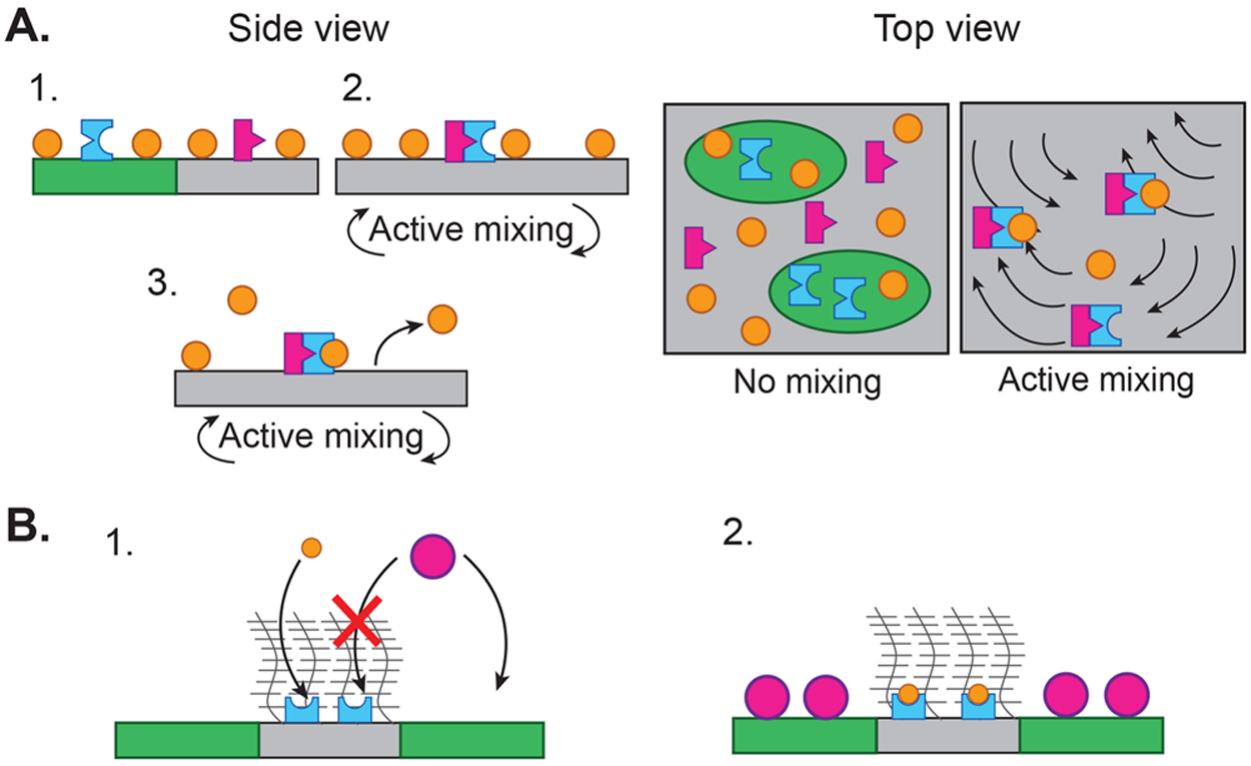
Applications of multifunctional “living” interfaces for surface binding and catalysis. (A) Active mixing from internally driven flows triggers the release of a phosphatidylcholine (PC)-bound substrate (orange) from a lipid bilayer. (*Left, panel 1*) Phospholipase D (PLD, blue) partitions into a liquid-ordered lipid domain (green), where it is physically kept separate from its PIP2 cofactor (magenta), preventing it from cleaving PC and releasing the substrate. (*Left, panel 2*) Active, internally driven mixing dissolves the domains, leaving a single-phase bilayer in which PLD and PIP2 are free to interact. (*Left, panel 3*) Having been activated by PIP2, PLD cleaves PC, releasing the orange substrate from the surface, into bulk solution. (*Right*) Top view, showing the dissolution of domains and interaction of PLD and PIP2 upon mixing. Active mixing triggers substrate release from the surface. (B) (*Panel 1*) A polymer brush adsorbed to a single lipid phase (gray) selectively prohibits a magenta contaminant from binding to the surface while admitting the smaller orange substrate to bind to a blue surface enzyme. The contaminant may freely bind to the green phase. (*Panel 2*) The contaminant and reactive components segregate onto different regions of the bilayer.

“Uncaging” membrane-bound substrates via the enzyme phospholipase D (PLD) offers a practical application for membrane-bound enzymatic reactions triggered via active mixing. PLD is activated by the lipid headgroup phosphatidylinositol 4,5-bisphosphate (PIP2) to hydrolyze phosphatidylcholine (PC), another common phospholipid headgroup, into a phosphatide and choline.^69^ It has been shown that, while PLD and PIP2 are normally physically segregated into distinct domains on native plasma membranes, mechanically shearing the cells can mix the domains, allowing the reaction to proceed when PLD meets its PIP2 cofactor.^69^ We propose developing a bio-enabled membrane surface with phase-separated lipid domains physically segregating PIP2 and PLD (Figure 5A). By infusing the membrane with PC lipids, functionalized with a chemical species of interest, such as a fluorophore or drug, we can effectively “cage” a chemical species on the membrane to be released into the bulk when PLD hydrolyzes PC. Triggering mixing via a surface-adsorbed actomyosin cortex “uncages” the adsorbed species, releasing it into bulk solution to perform a function, perhaps delivering a drug or reporting an analytical signal. While the system we present in Figure 3 remains as distinct phases upon active mixing, it has been shown that microtubules and kinesin can trigger mixing of liquid–liquid phase-separated DNA nanostar^70^ or PEG/dextran^40^ systems, and we expect this would be possible in 2D with the right lipid mixture and activity conditions.

Most surface materials are designed to perform a single task, such as gas exchange or chemical catalysis, and they often have a chronic problem of surface fouling by foreign contaminants. Preventing the fouling of surfaces from foreign contaminants like microbes, proteins, and particulate matter is a fundamental challenge in surface science and engineering.^71−73^ One can decorate membrane surfaces with self-driven biopolymers to generate autonomous surface flows that direct contaminant transport without externally imposed fluid flows or fields, overcoming a key challenge in fluid mechanics and surface science. Recent efforts in this area have focused on engineering synthetic active surfaces that transport cargo by mimicking ciliary clearance in the lungs.^36,74^ Combining these features on a single bio-enabled surface, such as a membrane with internally driven cytoskeletal filaments and a semipermeable protein brush, may enhance transport and reaction rates of desired species while preventing binding by inhibitory ligands (Figure 5B).

There has been considerable research interest in artificial cell membranes for many years, with many exciting developments in membrane vesicles that house DNA and RNA for protein and lipid synthesis and artificial organelles whose membranes adopt sponge morphologies reminiscent of the endoplasmic reticulum.^75,76^ Many of these vesicles are even mechanically active, with examples using lipid synthesis, osmotic pressure regulation, or actin ring contraction to drive membrane deformation and fusion.^75,76^ Furthermore, actin gliding assays have been reconstituted on planar membranes, displaying flocking and swarming behaviors, but without additional interaction with complex membrane components.^77^ However, the work we present here describes active matter dynamically mixing and reorganizing the constituent elements of lipid membranes in vitro. The active mixing we observe in Figure 3 complements chemical or other functions that a membrane might serve, controlling the spatial distribution and interaction frequency of membrane proteins and lipid species. Including additional elements like a glycocalyx-mimetic brush might add additional dimensions of filtering adsorbates and spatially tuning substrate binding, which are typically not seen in single-function artificial membranes. With the addition of these orthogonal, complementary functions, we expect the “living” bio-enabled materials we describe here to enhance the effectiveness of existing cell-free membrane devices and to enable further development of multifunctional surfaces.

In this Perspective, we have presented a very early realization of a bio-enabled “living” interface, in which mechanical activity mixes and drives coarsening in phase-separated lipid domains. However, many challenges remain before our model system can be realized in a practical material. We present a planar, surface-adsorbed membrane because it is most favorable for fluorescence microscopy analysis (Figure 2), but this may not be the most efficient geometry in applications where a high surface area:volume ratio is desired, such as surface catalysis. Applying actin to different membrane geometries, such as spherical vesicles, which can be mixed in a reactor, may prove to be a challenge, as actin is known to deform lipid membranes.^78^ Furthermore, myosin motors may deform the lipid membranes upon triggering activity without the presence of a solid support. Ultimately, if a solid support is deemed necessary to retain membrane integrity, coating the membrane on porous silica particles may enable these materials to retain their “living” nature while also maximizing their surface area.

Multifunctional materials offer an exciting opportunity to achieve orthogonal but complementary design goals in a single material system. Bio-enabled active matter surfaces may benefit product formulation, surface catalysis, drug delivery, antifouling, and other applications as they enable internal control of mechanical properties, biomolecular makeup, and spatial patterning of reactivity. Our group approaches these challenges by developing molecular probes to better understand the organization of living systems, with the intent of applying the findings to develop soft materials with unusual physical and chemical properties. We have discussed a few recent examples of work that our group and others in the field have addressed in understanding the spatial organization of native membranes and in developing model bio-enabled materials using purified proteins and lipids. A number of outstanding challenges remain in these approaches, but recent works from a number of laboratories show significant promise in the use of bio-enabled interfacial materials.
